# Measuring Dynamics in Evacuation Behaviour with Deep Learning

**DOI:** 10.3390/e24020198

**Published:** 2022-01-27

**Authors:** Huaidian Hou, Lingxiao Wang

**Affiliations:** 1 The Haverford School, 450 Lancaster Avenue, Haverford, PA 19010, USA; huaihou@haverford.org; 2 Frankfurt Institute for Advanced Studies, Ruth-Moufang-Str. 1, 60438 Frankfurt am Main, Germany; 3 Institute of Physics, Goethe-University Frankfurt, Max-von-Laue-Str. 1, 60438 Frankfurt am Main, Germany

**Keywords:** deep learning, crowd behaviour, evacuation

## Abstract

Bounded rationality is one crucial component in human behaviours. It plays a key role in the typical collective behaviour of evacuation, in which heterogeneous information can lead to deviations from optimal choices. In this study, we propose a framework of deep learning to extract a key dynamical parameter that drives crowd evacuation behaviour in a cellular automaton (CA) model. On simulation data sets of a replica dynamic CA model, trained deep convolution neural networks (CNNs) can accurately predict dynamics from multiple frames of images. The dynamical parameter could be regarded as a factor describing the optimality of path-choosing decisions in evacuation behaviour. In addition, it should be noted that the performance of this method is robust to incomplete images, in which the information loss caused by cutting images does not hinder the feasibility of the method. Moreover, this framework provides us with a platform to quantitatively measure the optimal strategy in evacuation, and this approach can be extended to other well-designed crowd behaviour experiments.

## 1. Introduction

As one of collective behaviours under extreme conditions, the crowd congestion in case of emergencies is routinely related to disasters, such as clogging stampede [1,2,3]. It becomes significant to investigate collective patterns and individual behaviours in such cases. Furthermore, relevant researches could also help bridge the gap between individual decisions and collective behaviours under extreme conditions. To achieve this, many researchers have been investigating collective behaviours in simulations and experiments for decades [1,2,3,4,5]. In the evacuation scenario, irrational behaviours are inevitable in decision-making [6,7,8], in which diverse behaviour patterns emerge [9]. In principle, “rationality” could describe an optimal strategy that will bring a maximum payoff at both individual and whole levels in game theory [10,11], which can be quantified by, e.g., minimizing escape time in evacuation behaviour [8,12]. Henceforth in the paper, we try to measure the optimality of exit decisions related to escape time in evacuation.

Without sufficient information from environments or enough capacities in a close space, optimality of path-choosing decisions made by each individual will also depend on others’ decisions, which can lead to the deviation from optimum strategies or generally introduce heterogeneous decision-makers [13,14,15,16,17,18]. Here, the deviation from optima is highly related to strategies of processing information [11,19,20,21], which is regarded as one possible origin of the bounded rationality [10]. In our study, we specify one of crucial factors as processing heterogeneous information [12,22,23,24,25]. It includes environment-related attributes and dynamics of surrounding pedestrians [6,26,27]. To combine them into a concrete case, we established a simulation model to describe a typical evacuation behaviour in a close space [12]. Although many works were applying macroscopic or microscopic models to study the evacuation behaviours [2,9,28,29,30], to evaluate individual strategies, micro-models represented by Social force model, CA model, and magnetic field force model [31,32,33] could give a more accurate description of individual behaviours [34,35,36,37,38]. Thus, to measure the deviation from optimum decisions, we further build a deep learning framework based on the CA evacuation simulation, in which the optimality of exit decisions is quantified as a dynamical parameter in our CA simulations [12], and more details are shown in Section 2.1.

**Figure 1:**
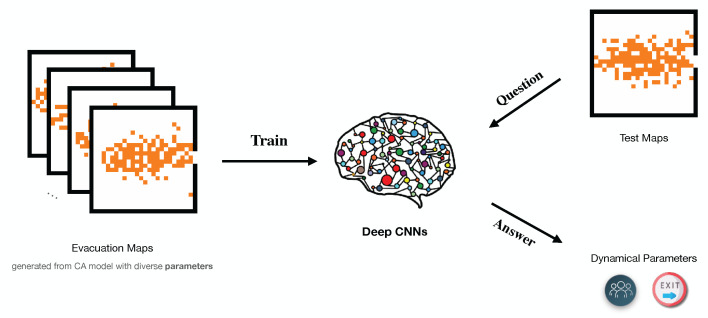
Flowchart of learning dynamical parameters in evacuation models and estimate their values in other cases. The left panel shows evacuation maps simulated from a CA model and they are inputs of a neural network. The middle panel represents a generic neural network model which can be specified as a deep CNN model in our case. The rightest panel contains two parts, in which the upper one is testing maps which is from simulations but can be conveniently extended to real observed images, and the bottom icons indicate the key dynamical information the well-trained neural network can predict. Here it is a rational factor but can be generalized to more dynamical parameters.

For the past few years, the development of sensor technology and the improvement of microchip computing power have been yielding unusually brilliant results in diverse fields. It makes things feasible that is collecting abundant data and using state-of-the-art machine learning methods to process them in evacuation behaviours [25,39,40,41,42,43]. Deep learning (DL), a branch of artificial intelligence (AI), efficiently integrates statistical and inference algorithms and thus offers opportunities to uncover hidden structures of evolution in complex data and to describe it with finite dynamical parameters. Therefore, a combination of DL algorithms and spatio-temporal models for evacuation based on bounded rationality is a promising option. The existing researches mainly focus on applications of DL in designing evacuation strategies based on data [44,45,46]. The other potential application is to train deep neural networks(DNNs) on simulated data sets and transfer them into real data sets to evaluate realistic situations or recognize the hidden signals, which has been verified in both physics and epidemiology [47,48,49]. Based on the methodology, as Figure 1 shows, we first introduce DL into the evacuation model to measure the optimality of decisions in such extreme scenes.

In this paper, we use replicator dynamics to simulate the evacuation, which combines the bounded rational behaviour and rational decision-making [11,12,50,51]. Adopting the simulation model proposed in Ref. [12], we deploy a DNN model to extract dynamical parameters that determine individual behaviour in a CA model describing evacuation behaviours. To train deep convolution neural networks (CNNs), we prepare data sets with various dynamical factors from multi-frame images generated by CA models, which specifically means training the deep CNN on images cut from the whole evolution process. In addition, this framework has been evaluated on four different CNN models and has been further examined on the data set consisting of incomplete images cut from original images.

## 2. Materials and Methods

### 2.1. Cellular Automaton Modeling Evacuation with Bounded Rationality

A cellular automaton model was proposed for simulating the pedestrian flow with bounded rationality in a two-dimensional system [12]. The underlying structure is a L×L cell grid, where *L* is system size. The state of a cell can be empty or occupied by one pedestrian exactly or wall. The Moore neighbor is adopted in CA models, and pedestrians update their positions by transition matrices P(i,t), where $P_{m,n}\left(i,t\right)$ means the possibility that pedestrian *i* moves from *t* time at position (x(i,t),y(i,t)) to next time-step position. Neighbors’ directions are labeled by (m,n), where m,n=1,2,3 represents the row and column index of 9 directions. Thus, (1,1) means the direction of upper left, (1,2) is the upper, (1,3) is the upper right and (2,2) represent the center and the others are defined in a similar fashion. Each cell could be either empty or occupied by a wall or a pedestrian. Pedestrians at each time step can choose to move into a new location or stop. Once we choose one location of the exit, the cellular automata updated synchronously can simulate the escape process [32,52].

The model escape rules gives as follows: Set the position of exit (x,y) and generate N(t) population distribution at a L×L lattice. At the t=0 time, disaster turns out and individuals begin to move; at the *t* time step, the individual *i* move to next position as matrices P(i,t) at t+1 time step. Update all individuals synchronously, and the conflict will be handled by compared transition possibilities; *Handle Conflicts*. The conflicts occur when two or more persons want to move into the same position, and what we do to handle the conflicts is to compare their transition possibilities $P_{m,n}\left(i,t\right)$ which reflects their willingness to move. For example, the individual *j* and *k* both want to move into position (x,y), and the corresponding possibility for *j* is $P_{m,n}\left(j,t\right)$ and *k* is $P_{m^{\prime },n^{\prime }}\left(k,t\right)$. If $P_{m,n}\left(j,t\right)>P_{m^{\prime },n^{\prime }}\left(k,t\right)$, then the individual *j* move successfully and *k* stayed where it was, and vice versa. For equal cases, one is randomly selected. It can be easily extended to the situation of many people. For individuals whose destination is an exit at the next time step, they escape successfully and are removed from the space to reduce the population as N(t+1)=N(t)−1. If N(t)=0, all individuals exited and stop evolution; Else, update transition matrix according to the above strategies.

The extreme situation of escaping from disasters constrains people’s behaviour, in which only intuition or social habits remains, no long term trade-off. The replicator dynamics modeling [50,51] links different behaviours, whether practical or spiritual, during a escaping process. It reforms the transition possibility P(i,t) as,

(1) $$P_{m,n}\left(i,t\right)=\frac{B_{m,n}\left(i,t\right)R_{m,n}\left(i,t\right)}{\sum B(i,t)R(i,t)}$$

where R(i,t),B(i,t) means weights from rational and bounded rational part respectively. They differ for different individual *i* at different time step *t*, which means these two matrices will be updated with evolution. The definition of components in matrix $R_{m,n}\left(i,t\right)=O_{m,n}\left(i,t\right)E_{m,n}\left(i,t\right)$,

(2) $$O_{m,n}\left(i,t\right)=\left\{\begin{array}{ccc} 1 & \; & empty\\ \epsilon & \; & occupied \end{array},E_{m,n}\left(i,t\right)=\{\begin{array}{ccc} \alpha & \; & exit\\ \epsilon & \; & nothing \end{array}\right.$$

which means if one position (m,n) around the individual *i* at *t* time is empty, $O_{m,n}\left(i,t\right)=1$, whereas the value is ϵ. And the $E_{m,n}\left(i,t\right)=\alpha$ only holds when the exit direction is indicated by (m,n). For each individual, there is a relative location of the exit, that location will be assigned into one of 9 directions mentioned before depending on which one has the smallest azimuth between the direction and the exit. The other directions take the value ϵ and the ϵ is a minimum value that the calculation accuracy can reach. The parameter α represents attraction of exit to persons who want to escape, or the importance of information of exit position in the model. As Ref. [12] shown, the increasing of parameter α will induce decreasing of escape time and eventually saturates at individual and system levels, which indicates that α is a potential indicator of measuring the optimality in evacuation behaviour. Thus, we named α as the rational parameter in such a CA model. To measure the optimality of path-choosing decisions in crowd behaviour is to extract the corresponding rational parameter α in our case.

The definition of bounded rational part $B_{m,n}$ relies on dynamic information from the others which leads to deviations from optima. The transport theory inspires us that escape dynamics needs more information on persons’ position and velocity distribution, the basic variables in transport theory. Considering the full information cannot easily be observed by individuals, the mean-field approximation (MFA) can provide a global perception for the people on move, which shows $B_{m,n}\left(i,t\right)=1$ as rational choices, $B_{m,n}\left(i,t\right)=n_{m,n}\left(i,t\right)$ as influencing by the crowds. The rational indicates transition possibilities only decided by R(i,t), that contains neighbours’ states and the direction of exit, or other objective environments. The crowd defines $n_{m,n}\left(i,t\right)=\sum_{m,n}N\left(i,t\right)/\sum_{All}N\left(i,t\right)$, where N(i,t) is the population distribution at *t* time. The definition shows the proportion of individuals in (m,n) orientation as a mean-field approximation, and people will be attracted to the direction with more density. We use it to mimic the “crowd” behaviour for individuals, which also means people can potentially get more population density information. The crowd effect induced by population affects human behaviour indirectly since people can gather and process information from the environment [22,25]. In this work, the distribution is discrete and the individual can process them as background, that’s what the above definition means. People’s perception of the distribution is reduced to the average value in a certain direction, a mean background field, as what statistical physics did in a many-body system.

### 2.2. Data-Set Generation and Network Capacity

The data sets that we prepared for training the neural networks are from the CA model included a total of 50,000 images. Out of the 50,000, there are 5 different initial populations $\rho_{0}$ ranging from 0.1 to 0.5, each with 10,000 images generated. Out of the 10,000 images with each initial population, there are 100 different values of rational parameters α∈(0,5) and 100 frames of evolution in Time-step T∈[1,100] for each parameter. Each image represents one snapshot of the evacuation process in a square form with a side length of 24, so each image we generated has 576 pixels. Each pixel of an image is either 0 or 1, where 0 represents empty space and 1 represents an individual present at that spot.

**Figure 2:**
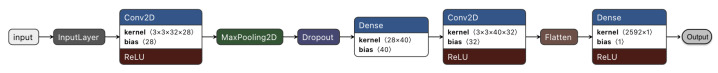
The CNN model we used to learn rational parameters from evolution. The fist module is the input layer which transfers 2-dimensional images into the following convolutional modules termed as Conv2D layers. A Conv2D layer includes 3×3 kernels and the ReLU activation function. The MaxPooling operation is only implemented after the first Conv2D layer and the Dropout is adopted to avoid the possible over-fitting. Between the final output layer and Conv2D layer, there is a Flatten operation to convert 2-dimensional intermediate data into a list which can be transferred by a fully-connected layer for output values. The fully-connected layer is named as Dense.

**Figure 3:**
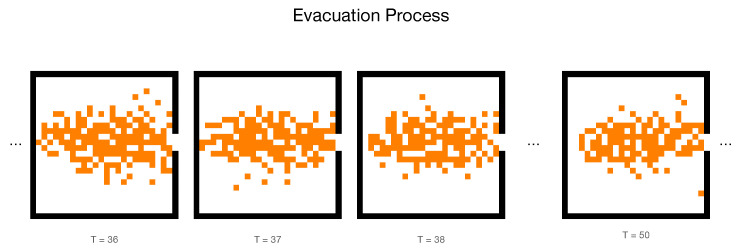
The simulation of an evacuation process with parameters $L=24,\alpha =10,\rho_{0}=0.37$. The orange sites represent the individuals and the white areas are empty spaces. The black solid line indicates the wall constrains the behaviours that occur in a closed room and the only exit locates at the rightest.

The main architecture of CNNs we used in this study is shown in Figure 2. Images generated from a CA model are fed to the input layer, the Conv2D layer is following after one input layer, and the MaxPooling layer is used to coarsening features extracted from CNN. The second Conv2D layer could be expanded to more CNNs whose performance is demonstrated in Section 3. The fully connected layers before the output layer are applied to process signals from preceding CNNs. The Droupout module and $L_{2}$ regularization are deployed to alleviate the possible over-fitting. To prepare inputs for the above CNN model, we select 10,000 as a standard batch size of samples, in which 2000 samples are from 5 initial population panels and mixed in one training data-set. Out of 2000, we label all 100 rational parameters α to each frame and prepared 20 groups from different frame selections. It means we prepare different numbers of consecutive frames as training data sets, which helps us to evaluate the performance of CNNs to extract the dynamical information from the collective behaviour. Starting with frame No. 36 as Figure 3 shown, we cut the following one frame as the first channel of 2000 samples, and then cut different numbers of frames (ranging from 1 to 32) after the first frame to form diverse channels of image inputs.

## 3. Results

### 3.1. Validating CNN Models

**Figure 4:**
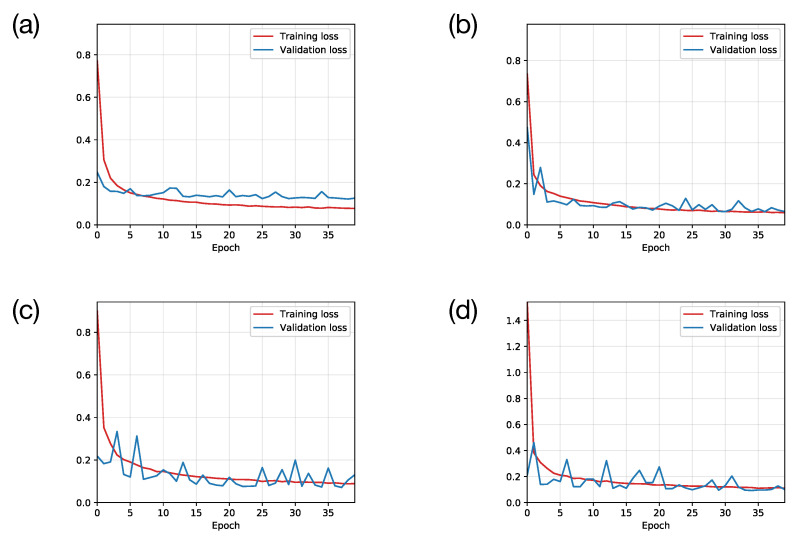
The training histories of the CNN models with 1, 2, 3 and 4 convolution layers correspond to (**a**–**d**). The blue lines are the loss on the validation data-set and the red line shows the corresponding loss on the training data-set.

To find a relative optimal CNN model to learn rational parameters from training data sets, we first examined the performance of different Convolution operations in our CNN models. In the examination, we set eight consecutive frames as eight channels per sample and tested different CNN models containing 1, 2, 3 and 4 convolution layers. The performances are demonstrated in Figure 4, in which the training and validation losses (mean square error, MSE) are decreasing with training. In Figure 4, the simple CNN model behaves distinct over-fitting after the first five epochs, which is understandable that the relative concise model tends to over-fit on a large data-set. Although the models with three and four convolution layers have small training losses as the other models show, their validation losses are highly unstable. It could be interpreted as the lack of training data causes under-fitting. The CNN model with two convolution layers is comparatively superior, for its stable performance both on training and validation data sets. Thus, in the following contents, we choose the 2-layer CNN model visualized in Figure 2 for further investigations.

### 3.2. Extracting the Dynamical Parameter via Deep Learning

**Figure 5:**
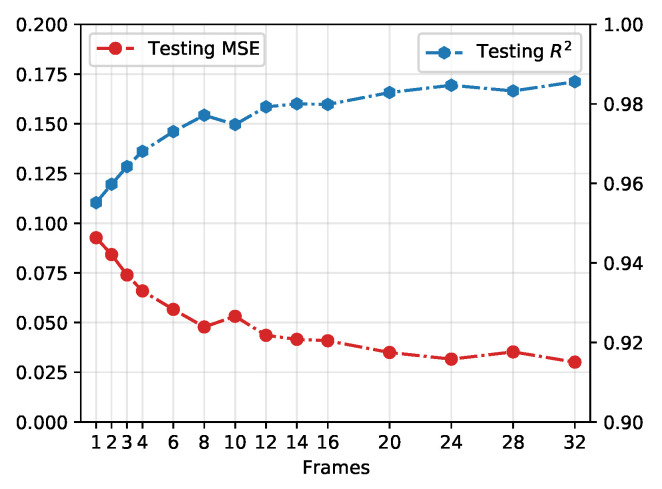
Testing performance on different numbers of consecutive frames. The blue dots are the correlation coefficients of the ground truth and predictions from the neural network. The red points indicate the corresponding mean square error on the testing data set.

In Figure 5, we demonstrate the testing performance of the CNN model on different numbers of consecutive frames. The MSE and $R^{2}=1-SS_{\mathrm{res}}/SS_{\mathrm{tot}}$ are chosen to evaluate the results learnt from different consecutive evolutions, where $SS_{\mathrm{res}}$ is the sum of squares of residuals between predictions and ground truths and $SS_{\mathrm{tot}}$ is the total sum of squares in testing data-set which is proportional to the variance of the data. By increasing the number of the frame from 1 to 32, the prediction of rational parameter $\alpha_{p}$ tends to reach the ground truth α. As a relatively ideal result, eight consecutive frames achieve a testing loss of 0.062 and R-squared value 0.9771. It should be mentioned that while increasing the frame number does increase overall accuracy by a marginal degree, the amount of data (here is time, in real-life applications) required to analyze in these models grows disproportionately against model accuracies. Selecting frame numbers as low as possible is more realistic for generalizing our framework to assist real-life applications to react more quickly.

To analyze sensitivity of the CNN model to extract rational parameter α under diverse population densities, we tested the CNN model on five initial population $\rho_{0}$ data-sets. To achieve the purpose, we prepare 2000 images from each $\rho_{0}$ using the same method as previously introduced, but here we feed images from each $\rho_{0}$ value into the model separately rather than mixed together. Five well-trained models are tested and shown in Table 1, in which results reveal that for all $\rho_{0}$ values we examined, the R-squared of evaluations were all above 0.98, while a 2000-image mixed model gives 0.95.

**Figure 6:**
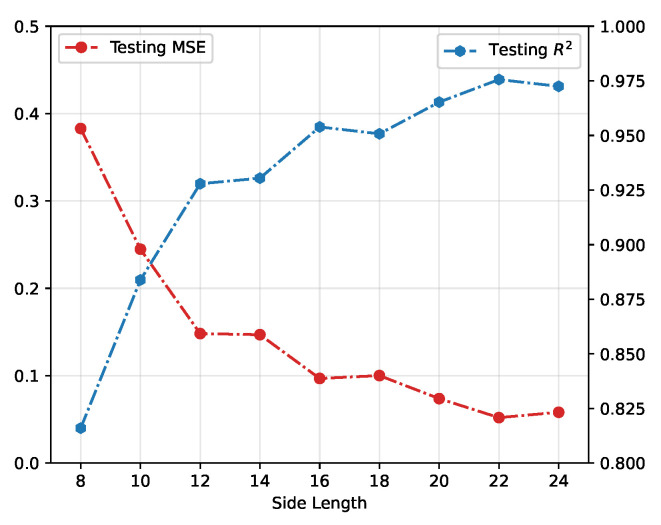
Testing performance on different side lengths of images cut from the top left corner. The blue dots are correlation coefficients of the ground truth and predictions from the neural network. The red dots indicate the mean square error on testing data set.

Now, we are concerned about a more realistic scenario, in which evacuation information is partially missing. In reality, the observations of collective behaviour are routinely noisy and(or) incomplete. How to process and understand the hidden behaviour patterns is one of the urgent topics in, e.g., stewardship [53] and social networks [54]. The CNN is trained on parts of the images we prepared before. This means that the side length and position of the prepared images are set to be different instead of the number of the frame. In concise, we select a square area with a given side length off from the 24 by 24 images we generated and determine the position of the image part by defining the coordinates of the upper left corner on the original image. Using the same 10,000 images and 2-layer CNN, the input images are set with side lengths from 8 to 24 (Images of side lengths less than 8 contain too little information to train a 2-layer CNN model). In Figure 6, results show that the longer the side length, the more accurate the prediction is, which is consistent with the information completeness. With regard to the position of cut images, the top left corner and right centre (where the exit is) are tested. The inspection using different side lengths as a sample shows the obvious advantage of providing information at the outlet. In addition, when monitoring the exit, a 12 by 12 image section can achieve an accurate prediction of the entire situation with MSE = 0.094 and $R^{2}=0.982$, which is close to the performance of training on complete images.

### 3.3. Robustness Examinations

**Figure 7:**
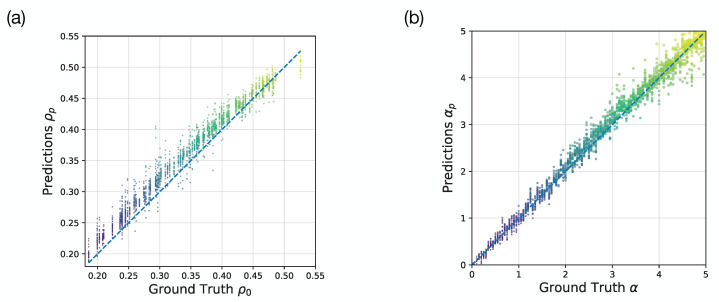
Robustness examinations. (**a**) The predicted initial population densities and their corresponding ground truths at the same dynamical factor α=5.0; (**b**) The predicted dynamical factors and their corresponding ground truths in the double-exit case at the same initial population density $\rho_{0}=0.5$. The colors of dots label the value of ground truths.

In addition to the dynamical factor α, other factors can also affect the evacuations, such as the initial population density and number of exits. In Figure 7, we validate our approach on these two cases. In Figure 7a, we use the same deep CNN model to learn the initial population densities from a series of intermediate processes which are (24,24,8) images. With the same size of data set, the testing performance is MSE = $6.73\times 10^{-4}$ and $R^{2}=0.906$. It achieves an acceptable performance, but not good as the prediction task to α. It is understandable that the only dynamical parameter of simulations is α which is more important than the initial condition for the intermediate processes. Concerning the double-exit case, we set two symmetric exits on both sides of the location in the single-exit case, and they have the same widths as the single one. In addition, under the same CNN model and size of data set, we get the testing performance as MSE = 0.053 and $R^{2}=0.973$. It is comparable to the single-exit case. The predicted α and ground truth are plotted in Figure 7b, they are consistent with each other.

### 3.4. Measuring Deviations from the Optimal Decision

With a well-trained CNN model, we can predict the rational parameter in such evacuation behaviour α that reflects the importance of the exit information to individuals. The crowd rule was introduced to characterize a bounded rational behaviour [12], in which the deviation from optima is measured in our framework. With the same processing as Section 2 to prepare data-sets, we generated 10,000 images under the crowd rule. In a transfer learning manner, the well-trained CNN model learnt on a data-set with optimal strategy is transferred to predict the rational parameters on the data-set with the crowd rule. As Table 2 shows, predictions of rational parameters on different initial population densities reveal a distinct deviation which is δα, in which the base-line α=2.475. The effect of the crowd rule on the rational behaviour is to reduce the influence of the exit information or equivalently is to strengthen the importance of the population density in evacuation. With population increasing, the deviation from optima δα changes from negative to positive, that is from overestimating rational parameter under small population to underestimating it under large population. In other words, the bounded rationality induced by the crowd rule in evacuation behaviour is quantitatively characterized as the deviation δα.

## 4. Conclusions

In this study, based on a CA model which generates spatio-temporal maps describing the evacuation process, we propose a deep learning framework to extract the optimality and its deviation induced by heterogeneous information. The latter is introduced in a replicator dynamics describing the bounded decision-making. The well-trained deep CNN accurately predicts dynamical factors from multi-frame images generated by the CA model. In addition, it should be noted that the performance of this machine is robust to incomplete images corresponding to global information loss.

This framework provides us with a platform in which the optimality of decision is measured as a dynamical parameter in evacuation simulations, and the latter can be simulated by replicator dynamics. It should be noted that deep CNN is just one of the machine learning approaches that can learn the dynamical factor from replicator dynamics. Besides, the Bayesian method can also achieve our goal [55,56]. Although the CNNs can capture the spatial correlations more naturally in image-type data, it still deserves to compare the performances of different methods in the future. Furthermore, the scheme could also be generalized to other well-designed experiments. It has potentials to be used in recognizing potential collective patterns and avoid trampling if we trained on observed image data-sets from experiments or the real world. On the other hand, combining online games with the deep learning framework, it can help us to measure the optimality of individual or group decision in more human behaviours [57,58,59,60]. Because the evacuation simulation provides us with a platform in which the human instinct dominates behaviours under extreme scenes [23,61,62]. It brings opportunities to effectively investigate human behaviours without complex social relations, which will help us to understand the diverse and fascinating collective behaviours that occur in both virtual and real space (social network, financial network and social norms, these virtual social connections naturally incubate the collective behaviour; as for the real space, collective modes are common in urban dynamics, traffic flow, and pedestrian dynamics [63,64]). An online game simulating multi-players in evacuation has been developed and the measurement results will be released in our future works. In summary, this study provides an insight into measuring human decisions with deep learning approaches in collective behaviours.

## Figures and Tables

**Table 1:** Test accuracy on different initial population densities.

$\rho_{0}$	0.1	0.2	0.3	0.4	0.5	Mixed
MSE	0.0150	0.0397	0.0134	0.0233	0.0095	0.0914
MAE	0.0387	0.0609	0.0401	0.0541	0.0289	0.2350
$R^{2}$	0.9881	0.9899	0.9910	0.9852	0.9913	0.9565

**Table 2:** Predictions of the dynamical parameter on different initial population densities.

$\rho_{0}$	0.1	0.2	0.3	0.4	0.5	Mixed
$\alpha_{p}$	2.514	2.509	2.445	2.424	2.348	2.448
δα	0.039	0.034	−0.020	−0.051	−0.127	−0.027

## Data Availability

Not applicable.

## References

[1] Helbing D., Farkas I., Vicsek T. (2000). Simulating Dynamical Features of Escape Panic. Nature.

[2] Hughes R.L. (2002). A Continuum Theory for the Flow of Pedestrians. Transp. Res. Part B Methodol..

[3] Helbing D., Buzna L., Johansson A., Werner T. (2005). Self-Organized Pedestrian Crowd Dynamics: Experiments, Simulations, and Design Solutions. Transp. Sci..

[4] Pastor J.M., Garcimartín A., Gago P.A., Peralta J.P., Martín-Gómez C., Ferrer L.M., Maza D., Parisi D.R., Pugnaloni L.A., Zuriguel I. (2015). Experimental Proof of Faster-Is-Slower in Systems of Frictional Particles Flowing through Constrictions. Phys. Rev. E.

[5] Nicolas A., Ibáñez S., Kuperman M.N., Bouzat S. (2018). A Counterintuitive Way to Speed up Pedestrian and Granular Bottleneck Flows Prone to Clogging: Can ‘more’ Escape Faster?. J. Stat. Mech..

[6] Wijermans F.E.H. (2011). Understanding Crowd Behaviour: Simulating Situated Individuals.

[7] Vermuyten H., Beliën J., De Boeck L., Reniers G., Wauters T. (2016). A Review of Optimisation Models for Pedestrian Evacuation and Design Problems. Saf. Sci..

[8] Haghani M., Sarvi M. (2019). ‘Rationality’ in Collective Escape Behaviour: Identifying Reference Points of Measurement at Micro and Macro Levels. J. Adv. Transp..

[9] Bain N., Bartolo D. (2019). Dynamic Response and Hydrodynamics of Polarized Crowds. Science.

[10] Simon H.A. (1983). Reason in Human Affairs.

[11] Pan Q., Wang L., Shi R., Wang H., He M. (2014). Spatial Modes of Cooperation Based on Bounded Rationality. Phys. A.

[12] Wang L., Jiang Y. (2019). Escape Dynamics Based on Bounded Rationality. Phys. A.

[13] Noh D.j., Koo J., Kim B.I. (2016). An Efficient Partially Dedicated Strategy for Evacuation of a Heterogeneous Population. Simul. Model. Pract. Theory.

[14] Dixon D.S., Mozumder P., Vásquez W.F., Gladwin H. (2017). Heterogeneity Within and Across Households in Hurricane Evacuation Response. Netw. Spat. Econ..

[15] Guo X., Chen J., Zheng Y., Wei J. (2012). A Heterogeneous Lattice Gas Model for Simulating Pedestrian Evacuation. Phys. A.

[16] Haghani M., Sarvi M. (2019). Heterogeneity of Decision Strategy in Collective Escape of Human Crowds: On Identifying the Optimum Composition. Int. J. Disaster Risk Reduct..

[17] Liu Q. (2018). The Effect of Dedicated Exit on the Evacuation of Heterogeneous Pedestrians. Phys. A Stat. Mech. Its Appl..

[18] Petrolia D.R., Bhattacharjee S., Hanson T.R. (2011). Heterogeneous Evacuation Responses to Storm Forecast Attributes. Nat. Hazards Rev..

[19] Gigerenzer G., Selten R. (2002). Bounded Rationality: The Adaptive Toolbox.

[20] Yang X., Yao S. (2005). Walrasian Sequential Equilibrium, Bounded Rationality, and Social Experiments. Div. Labor Trans. Costs.

[21] Băbeanu A.I., Garlaschelli D. (2018). Evidence for Mixed Rationalities in Preference Formation. Complexity.

[22] Lee K., Hui P.M., Wang B.H., Johnson N.F. (2001). Effects of Announcing Global Information in a Two-Route Traffic Flow Model. J. Phys. Soc. Jpn..

[23] Nowak A., Vallacher R.R., Zochowski M. (2005). The Emergence of Personality: Dynamic Foundations of Individual Variation. Dev. Rev..

[24] Wang W.X., Wang B.H., Zheng W.C., Yin C.Y., Zhou T. (2005). Advanced Information Feedback in Intelligent Traffic Systems. Phys. Rev. E.

[25] Moussaïd M., Helbing D., Theraulaz G. (2011). How Simple Rules Determine Pedestrian Behavior and Crowd Disasters. Proc. Natl. Acad. Sci. USA.

[26] Bode N.W.F., Kemloh Wagoum A.U., Codling E.A. (2014). Human Responses to Multiple Sources of Directional Information in Virtual Crowd Evacuations. J. R. Soc. Interface.

[27] Haghani M., Sarvi M. (2017). Social Dynamics in Emergency Evacuations: Disentangling Crowd’s Attraction and Repulsion Effects. Phys. A Stat. Mech. Its Appl..

[28] Low D.J. (2000). Statistical Physics: Following the Crowd. Nature.

[29] Nicolas A., Kuperman M., Ibañez S., Bouzat S., Appert-Rolland C. (2019). Mechanical Response of Dense Pedestrian Crowds to the Crossing of Intruders. Sci. Rep..

[30] Ma Y., Lee E.W.M., Shi M., Yuen R.K.K. (2021). Spontaneous Synchronization of Motion in Pedestrian Crowds of Different Densities. Nat. Hum. Behav..

[31] Helbing D., Molnár P. (1995). Social Force Model for Pedestrian Dynamics. Phys. Rev. E.

[32] Burstedde C., Klauck K., Schadschneider A., Zittartz J. (2001). Simulation of Pedestrian Dynamics Using a Two-Dimensional Cellular Automaton. Phys. A.

[33] Weng W.G., Chen T., Yuan H.Y., Fan W.C. (2006). Cellular Automaton Simulation of Pedestrian Counter Flow with Different Walk Velocities. Phys. Rev. E.

[34] Patterson G.A., Fierens P.I., Sangiuliano Jimka F., König P.G., Garcimartín A., Zuriguel I., Pugnaloni L.A., Parisi D.R. (2017). Clogging Transition of Vibration-Driven Vehicles Passing through Constrictions. Phys. Rev. Lett..

[35] Aguilar J., Monaenkova D., Linevich V., Savoie W., Dutta B., Kuan H.S., Betterton M.D., Goodisman M.A.D., Goldman D.I. (2018). Collective Clog Control: Optimizing Traffic Flow in Confined Biological and Robophysical Excavation. Science.

[36] Dressaire E., Sauret A. (2017). Clogging of Microfluidic Systems. Soft Matter.

[37] Delarue M., Hartung J., Schreck C., Gniewek P., Hu L., Herminghaus S., Hallatschek O. (2016). Self-Driven Jamming in Growing Microbial Populations. Nat. Phys..

[38] Garcimartín A., Pastor J.M., Ferrer L.M., Ramos J.J., Martín-Gómez C., Zuriguel I. (2015). Flow and Clogging of a Sheep Herd Passing through a Bottleneck. Phys. Rev. E.

[39] Helbing D., Tröster G., Wirz M., Roggen D. (2011). Recognition of Crowd Behavior from Mobile Sensors with Pattern Analysis and Graph Clustering Methods. Netw. Heterog. Media.

[40] Corbetta A., Lee C.M., Benzi R., Muntean A., Toschi F. (2017). Fluctuations around Mean Walking Behaviors in Diluted Pedestrian Flows. Phys. Rev. E.

[41] Zanlungo F., Yucel Z., Brscic D., Kanda T., Hagita N. (2017). Intrinsic Group Behaviour: Dependence of Pedestrian Dyad Dynamics on Principal Social and Personal Features. PLoS ONE.

[42] Wang C., Weng W. (2018). Study on the Collision Dynamics and the Transmission Pattern between Pedestrians along the Queue. J. Stat. Mech..

[43] Tordeux A., Chraibi M., Seyfried A., Schadschneider A. (2020). Prediction of Pedestrian Dynamics in Complex Architectures with Artificial Neural Networks. J. Intell. Transp. Syst..

[44] Rahman R., Hasan S. Short-Term Traffic Speed Prediction for Freeways During Hurricane Evacuation: A Deep Learning Approach. Proceedings of the 2018 21st International Conference on Intelligent Transportation Systems (ITSC).

[45] Song X., Shibasaki R., Yuan N.J., Xie X., Li T., Adachi R. (2017). DeepMob: Learning Deep Knowledge of Human Emergency Behavior and Mobility from Big and Heterogeneous Data. ACM Trans. Inf. Syst..

[46] Chen Y., Hu S., Mao H., Deng W., Gao X. (2020). Application of the Best Evacuation Model of Deep Learning in the Design of Public Structures. Image Vis. Comput..

[47] Pang L.G., Zhou K., Su N., Petersen H., Stöcker H., Wang X.N. (2018). An Equation-of-State-Meter of Quantum Chromodynamics Transition from Deep Learning. Nat. Commun..

[48] Jiang L., Wang L., Zhou K. (2021). Deep Learning Stochastic Processes with QCD Phase Transition. Phys. Rev. D.

[49] Wang L., Xu T., Stoecker T., Stoecker H., Jiang Y., Zhou K. (2021). Machine Learning Spatio-Temporal Epidemiological Model to Evaluate Germany-county-level COVID-19 Risk. Mach. Learn. Sci. Technol..

[50] Heliövaara S., Ehtamo H., Helbing D., Korhonen T. (2013). Patient and Impatient Pedestrians in a Spatial Game for Egress Congestion. Phys. Rev. E.

[51] Taylor P.D., Jonker L.B. (1978). Evolutionary Stable Strategies and Game Dynamics. Math. Biosci..

[52] Kirchner A., Nishinari K., Schadschneider A. (2003). Friction Effects and Clogging in a Cellular Automaton Model for Pedestrian Dynamics. Phys. Rev. E.

[53] Bak-Coleman J.B., Alfano M., Barfuss W., Bergstrom C.T., Centeno M.A., Couzin I.D., Donges J.F., Galesic M., Gersick A.S., Jacquet J. (2021). Stewardship of Global Collective Behavior. Proc. Natl. Acad. Sci. USA.

[54] Stewart A.J., Mosleh M., Diakonova M., Arechar A.A., Rand D.G., Plotkin J.B. (2019). Information Gerrymandering and Undemocratic Decisions. Nature.

[55] Strelioff C.C., Crutchfield J.P., Hübler A.W. (2007). Inferring Markov Chains: Bayesian Estimation, Model Comparison, Entropy Rate, and out-of-Class Modeling. Phys. Rev. E.

[56] Strelioff C.C., Crutchfield J.P. (2014). Bayesian Structural Inference for Hidden Processes. Phys. Rev. E.

[57] Mao A., Mason W., Suri S., Watts D.J. (2016). An Experimental Study of Team Size and Performance on a Complex Task. PLoS ONE.

[58] Van Dolder D., van den Assem M.J. (2018). The Wisdom of the Inner Crowd in Three Large Natural Experiments. Nat. Hum. Behav..

[59] Awad E., Dsouza S., Kim R., Schulz J., Henrich J., Shariff A., Bonnefon J.F., Rahwan I. (2018). The Moral Machine Experiment. Nature.

[60] Toyokawa W., Whalen A., Laland K.N. (2019). Social Learning Strategies Regulate the Wisdom and Madness of Interactive Crowds. Nat. Hum. Behav..

[61] Nicolas A., Garcimartín Á., Zuriguel I. (2018). Trap Model for Clogging and Unclogging in Granular Hopper Flows. Phys. Rev. Lett..

[62] Cavagna A., Giardina I., Grigera T.S. (2018). The Physics of Flocking: Correlation as a Compass from Experiments to Theory. Phys. Rep..

[63] Castellano C., Fortunato S., Loreto V. (2009). Statistical Physics of Social Dynamics. Rev. Mod. Phys..

[64] Ball P. (2012). Why Society Is a Complex Matter: Meeting Twenty-First Century Challenges with a New Kind of Science.

